# Consensus Statements for Assessment and Management of Threatened Miscarriage in the First Trimester in Pakistan: A Three-Step Modified Delphi Approach

**DOI:** 10.7759/cureus.65079

**Published:** 2024-07-22

**Authors:** Maryam Iqbal, Maryam Zubair, Azra Saeed Awan, Yousaf Khan, Haleema Yasmin, Rehana Rahim, Pushpa Srichand, Sadiah A Pal, Syeda Batool Mazhar, Rubina Sohail, Farrukh Zaman, Sobia Ali, Tabrez Ali

**Affiliations:** 1 Obstetrics and Gynecology, Integrated Medical Care Hospital, Lahore, PAK; 2 Obstetrics and Gynecology, Azad Jammu Kashmir Medical College, Muzaffarabad, PAK; 3 Obstetrics and Gynecology, Fauji Foundation Hospital, Rawalpindi, PAK; 4 Obstetrics and Gynecology, Hameed Latif Hospital, Lahore, PAK; 5 Obstetrics and Gynecology, Jinnah Postgraduate Medical Center (JPMC), Karachi, PAK; 6 Obstetrics and Gynecology, Lady Ready Hospital, Peshawar, PAK; 7 Obstetrics and Gynecology, Isra University Hospital, Hyderabad, PAK; 8 Epidemiology, Concept Fertility Center, Karachi, PAK; 9 Obstetrics and Gynecology, Pakistan Institute of Medical Sciences, Islamabad, PAK; 10 Epidemiology and Public Health, Hameed Latif Hospital, Lahore, PAK; 11 Medical Affairs, Established Pharmaceutical Division, Abbott Laboratories (Pakistan) Limited, Karachi, PAK

**Keywords:** vaginal bleeding, miscarriage, consensus, pakistan, threatened miscarriage

## Abstract

Background and objective: It aimed to develop an expert consensus regarding the risk assessment, diagnosis, and threatened miscarriage management during the first trimester in Pakistan.

Methods: A three-step modified Delphi method was applied to develop the consensus. Eleven specialized obstetricians and gynecologists participated in its development. If 75% or higher agreement level was attained on each assertion, it was declared as a consensus.

Results: Age of 35 or above, previous history of two or more previous miscarriages, and direct strong trauma were considered to be threatened miscarriage risk factors. Infection was discussed and specified to include specific infectious diseases, like malaria, and COVID-19 as a risk factor. The experts agreed from the first time on considering endocrinological disorders, thrombophilia, and lifestyle variables as threatened miscarriage risk factors. They proposed adding a statement concerning acquired thrombophilia which was accepted unanimously. Finally, experts agreed on the importance of educating pregnant women about factors whose risk can be modified by modifying their behavior. As for diagnosis statements, it was agreed to be trifold: physical examination, imaging, and laboratory testing. Physical examination included abdominal and pelvic exams but focused more on vaginal examination with speculum to identify bleeding severity and etiology. The statements regarding the imaging approaches to diagnose threatened miscarriage in the first trimester achieved a consensus in most statements. TVS was recommended to check on uterine structural abnormalities, fetus viability focusing on heartbeat and crown-to-rump length, gestation sac size and emptiness, subchorionic hematoma, and ectopic pregnancy. Each was defined on how to identify and diagnose in separate statements. Statements about laboratory tests indicated the need for human chorionic gonadotropin hormone assessment whether serial or once is dependent on the ultrasound. Recommended hematologic investigations include complete blood count for anemia, Rh factor for potential bleeding risk and in special cases, thrombophilia assessment is undertaken. The first and foremost management aspect was follow-up while most management statements were controversial, and some were altogether removed with only some reaching agreement after discussion.

Conclusion: These consensus statements aggregated the best available evidence and experts’ opinion-supported statements to improve patient education, risk assessment, diagnosis, and evaluation as well as management of threatened miscarriage during the first trimester in Pakistan.

## Introduction

As defined by the World Health Organization, “threatened miscarriage is pregnancy related bloody vaginal discharge or frank bleeding during the first half of pregnancy without cervical dilatation” [1]. Threatened miscarriage is determined by vaginal bleeding occurring within 20 weeks of gestation along with a positive pregnancy test and a cl osed cervical os [1,2].

Threatened miscarriage affects about 20% of pregnant women and is considered the most common complication of early pregnancy [3]. Despite the fact that many women who suffer from threatened miscarriage can have a successful pregnancy; there is a 2.6-fold increase in the risk of miscarriage in the same pregnancy [4]. About 17% of pregnant women with threatened miscarriages go on to have additional complications in the same pregnancy [5,6]. However, about a quarter of pregnant women with vaginal bleeding have more than 10% subsequent risk of miscarriage [7]. The National Institute of Population Studies in Pakistan 2006-2007 have stated that the percentage of miscarriages among married women in Pakistan was around 8% [8]. Yet, a study in Pakistan showed an increase in the percentage of miscarriages from 6% to 12% between 2015 and 2020 [9].

During a threatened miscarriage, the bleeding is usually mild to moderate with or without abdominal pain presented as intermittent cramping, suprapubic pain, pelvic pressure, or lower back pain [1]. Early pregnancy pain and bleeding affect roughly one out of every five clinically confirmed pregnancies. The pregnancy will continue and have a successful outcome in 50%-60% of these cases, but the symptoms may indicate impending miscarriage (25%-30%) or ectopic pregnancy (10%-15%) [6].

Threatened miscarriage has been linked to an increased risk of obstetric complications like Antepartum hemorrhage (APH), preterm delivery, cesarean delivery, preeclampsia, placenta previa, and placenta abruption, as well as adverse perinatal outcome measures such as small for gestational age and early neonatal death [10,11].

The necessity of identifying risk factors for threatened miscarriage plays a major role in the condition's prevention and treatment. Age, obesity, stress level, past miscarriage history, and the existence of medical conditions are all known risk factors [12].

Since the incidence of miscarriage is more than 10% and given the significant impact of miscarriage on women's physical and mental health, the couple's financial and social standing, and the Pakistani healthcare system, it is crucial to develop a consensus to assist obstetricians and gynecologists in diagnosing and managing threatened miscarriage. Although some references and guidelines were found dealing with threatened miscarriage, no consensus was noted in Pakistan [13]. This consensus is based on identifying and critically appraising research evidence and generating recommendations based on a combination of the available evidence and expert opinion in Pakistan.

A Delphi method-based study was conducted to develop a consensus regarding the assessment, evaluation, and management of threatened miscarriage during the first trimester as well as the follow-up and education implications of threatened miscarriage during the first trimester in pregnant women in Pakistan. A three-step Delphi survey was adopted to integrate the opinions of experts and to develop the final form of the guidelines to assess, evaluate, and manage threatened miscarriage during the first trimester in pregnant women in Pakistan.

## Materials and methods

Study design

The three-step Delphi method to develop a consensus concerning risk assessment, diagnosis, and management of threatened miscarriage in Pakistan. Fifty-one consensus statements were developed and followed by two rounds of anonymous voting and a virtual expert discussion meeting where the different statements were voted on and discussed for inclusion in the final consensus.

Expert panel recruitment

Eleven experts specializing in obstetrics and gynecologists from Pakistan were involved in the consensus. All experts had an active research profile and were either affiliated with an academic institution, a public sector hospital, or a private sector hospital in Pakistan. Eligible experts were invited to participate in the Delphi study through email and were asked to complete all three steps of voting and meeting. Statements reach a consensus if the voting agreement was more than 70% among experts.

Survey development

A systematic literature search of Medline via PubMed, UpToDate, and Cochrane Database of systematic reviews, randomized controlled trials, non-randomized controlled trials, and cohort studies, and international guidelines was conducted from each database’s inception date till January 2022. This search aimed to collect relevant information from the survey development committee concerning threatened miscarriage literature. The main keywords and Medical Subject Heading terms used in the literature search were the following: “abortion, threatened” (MeSH Terms), “threatened abortion,” “threatened miscarriage,” “miscarriage,” “abortion, spontaneous” (MeSH Terms), “miscarriage,” “pregnancy loss,” “vaginal bleeding,” “pregnancy trimester, first” (MeSH Terms), “first-trimester pregnancy,” “first trimester bleeding.” Initially, the titles and abstracts of the retrieved records were screened. Then, full texts of the publications that address the consensus objectives were also screened. A manual search of the references of retrieved publications was also conducted. Furthermore, we screened the guidelines of major international societies, including the National Health Institute (NIH), National Institute for Health Care and Excellence (NICE), American College of Obstetricians and Gynecologists (ACOG), Society of Obstetrician and Gynecologists of Canada (SOGC), Mayo Clinic, and American Pregnancy Association for additional statements. The statements were retrieved from studies and ranked according to the GRADE system's “high, moderate, low” levels of evidence [14].

## Results

Health-related risk factors

The statements related to health risk factors are outlined in Table 1. All experts agreed on the major risk factors for miscarriage, including bacterial, viral, parasitic, or fungal infections and their management.

**Table 1:** Level of agreement on statements concerning health-related risk factors

Statements	Level of agreement N(%)
Severe infections such as viral and parasitic (Malaria, Dengue, COVID) leading to septicemia or viremia may increase the risk of and are associated with threatened miscarriage. (rephrased).	11(100)
Endocrine factors and metabolic abnormalities such as diabetes, polycystic ovary syndrome and abnormal thyroid function (hypothyroidism) are considered risk factors for threatened miscarriage.	11(100)
The effect of inheritable thrombophilia on pregnancy loss risk is unclear as the body of evidence conflicts.	9(81.8)
Acquired thrombophilia is considered a risk factor for threatened miscarriage.	11(100)

Lifestyle-related risk factors

Statements related to lifestyle risk factors are outlined in Table 2. It consists of three statements. About 91% of the experts agreed to consider smoking, caffeine, alcohol, and illicit drug use as risks for threatened miscarriage with dose-related factors for smoking, caffeine, and alcohol consumption. Also, all experts agreed that being overweight or underweight is considered a risk factor for threatened miscarriage.

**Table 2:** Level of agreement on the statements concerning lifestyle-related risk factors

Statement	Level of agreement (%)
Being overweight or underweight are considered risk factors for threatened miscarriage.	11(100)
Smoking, caffeine, and alcohol consumption are considered risk factors for threatened miscarriage in a dose/related fashion.	10(90.9)
Illicit drug use (cocaine or methamphetamines) is considered a risk for pregnancy loss in threatened miscarriage.	10(90.9)

Physical examination

Statements related to physical examination to diagnose a threatened miscarriage are outlined in Table 3. Experts agreed on the physical examination process.

**Table 3:** Level of agreement on statements concerning physical examination used to diagnose threatened miscarriage

Statement	Level of agreement (%)
Abdominal examination should be performed before the internal examination. It is best to begin by examining the quadrant where the patient is experiencing the least pain. (Rephrased)	11(100)
Non-gynecologic causes of pain, such as gastrointestinal, should also be considered while performing the abdominal examination.	10(90.9)
Pelvic examination and determination of uterine size with respect to gestational age should be identified if facility of ultrasound is not available.	11(100)
Internal vaginal examination is recommended to be done by inserting a speculum into the vagina to assess the volume and source of bleeding. If blood clots are present, they can be removed with gauze sponges on sponge forceps.	11(100)
Speculum examination is recommended as it may reveal a source of bleeding unrelated to pregnancy (i.e. vaginal laceration, vaginal warts, cervical polyps, fibroids...); in such cases, further evaluation depends upon the nature of the abnormality.	10(90.9)
The source of bleeding should be investigated to exclude extra-vaginal bleeding.	9(81.8)
It is recommended to check the cervix, if closed or not, and for any abnormality such as cervical erosion, polyps, and inflammation by internal vaginal examination. A closed internal cervical os is most consistent with threatened miscarriage, but not diagnostic.	10(90.9)
Direct visualization of the gestational sac in a dilated internal cervical os is generally sufficient to conclude that early pregnancy loss is inevitable.	9(81.8)
A vaginal swab is recommended in the presence or suspicion of vaginal infections.	11(100)
HVS (High Vaginal Swab for culture sensitivity) is recommended in the presence or suspicion of vaginal infection.	11(100)

Imaging approaches

Statements related to imaging approaches to diagnose threatened miscarriage are outlined in Table 4. The experts agreed that Transvaginal ultrasonography (TVS) is recommended in checking for structural abnormalities of the uterus, evidence of viability and intrauterine location, the possibility of extrauterine pregnancy or heterotopic pregnancy, and loss of one of the multiple gestations.

**Table 4:** Level of agreement on statements concerning imaging approaches used to diagnose threatened miscarriage in the first trimester in Pakistan

Statement	Level of agreement (%)
Transvaginal ultrasonography is recommended to	
- Check structural abnormalities of the uterus.	11(100)
- Confirm both an intrauterine gestation and evidence of viability.	10(90.9)
- Check the possibility of EP/heterotopic pregnancy and the loss of one gestation from a multiple gestation.	11(100)
- Help in determination of chorionicity in case of multiple gestations.	11(100)
Sonography/ultrasound examination is the recommended modality to check for fetal heart activity and to assess the discrepancy between gestational age and crown to rump length.	11(100)
Sub-chorionic hematomas are common and are present in up to 18% of women who present with a threatened miscarriage. They are insignificant sonographic findings and there is no association between the size of hematoma and the rate of threatened miscarriage.	11(100)
If the uterus contains an empty gestational sac ≥ 25 mm on TVS, this is almost certainly an anembryonic pregnancy; however, a scan repeated at an interval of 7 days is recommended if there is any doubt.	11(100)
EP can be suspected/ diagnosed when an adnexal mass is detected separately from the ovaries.	11(100)
The diagnosis of EP should not be based on an inability to visualize an intrauterine pregnancy (IUP), but rather on the positive visualization of an adnexal mass using TVS.	10(90.9)
Embryonic death can be diagnosed when ultrasound imaging shows an intrauterine gestational sac, embryonic crown-rump length ≥ 7 mm, no cardiac activity.	9(81.8)
Anembryonic pregnancy or early embryonic death can be diagnosed when the diameter of the gestational sac is ≥ 25 mm without a yolk sac or embryo.	11(100)

The panel agreed that Sonography/ultrasound examination is the cornerstone to check heartbeats and if there is fetal bradycardia then they should be referred to a specialist. Since the prognostic value of a subchorionic hematoma in ultrasound has been disputed, there was a unanimous agreement that it has no association with threatened miscarriage. All other statements were undertaken during the discussion and agreed upon with the majority being approved unanimously.

Laboratory tests

Statements related to the value of laboratory tests are outlined in Table 5. This section includes six statements. All experts agreed on the tests needed to diagnose threatened miscarriage in the first trimester in Pakistan.

**Table 5:** Level of agreement on statements concerning laboratory tests to diagnose threatened miscarriage in the first trimester in Pakistan

Statement	Level of agreement (%)
There is no role for monitoring hCG concentration once the presence of an intrauterine pregnancy has been established sonographically. It is advised that serial measurements of hCG are helpful during the first six weeks of pregnancy if ultrasonography is nondiagnostic.	11(100)
Failure to detect an intrauterine gestational sac, by transvaginal ultrasound, when the β-hCG value exceeds a discriminatory level of 2,000–3,000 mIU/mL indicates an increased risk for EP.	11(100)
A combination of serial human serum hCG levels and serial ultrasound examinations are recommended to exclude ectopic pregnancy. A serum beta-hCG level of 1,500 lU/mL to 2,000 lU/mL is associated with a gestational sac on ultrasound.	11(100)
Rh factor testing should be performed if Rh status is not known at the time of presentation.	10(90.9)
Complete blood Count (CBC) should be performed to assess the degree of blood loss and anemia.	9(81.8)
Women with recurrent miscarriage can be offered thrombophilic blood screening and be referred to specialist.	11(100)

Management of threatened miscarriage in the first trimester

Statements related to the management of threatened miscarriage are outlined in Table 6.

**Table 6:** Level of agreement on statements concerning the management of threatened miscarriage in Pakistan

Statement	Level of agreement (%)
Patients with a threatened miscarriage should be managed expectantly without any medical or surgical interventions. However, patients should be given strict return precautions concerning excessive vaginal bleeding, abdominal pain, or fever and patients should be educated on the importance of follow up.	11(100)
In clinically stable or asymptomatic patients, when suspicion of early pregnancy loss is being considered, a follow-up ultrasound scan should be booked after an additional 7–10 days until a viable intrauterine pregnancy is confirmed or until progression to an inevitable, incomplete, or complete abortion occurs.	11(100)
In case of pain, analgesia can be provided to help relieve discomfort from cramping. NSAIDs should be avoided in the setting of threatened miscarriage.	11(100)
Dydrogesterone 40 mg stat dose followed by 10 mg twice a day for one week or conservative therapy has been proven to reduce the incidence of pregnancy loss in threatened miscarriage during the first trimester.	11(100)
Missed miscarriage can be defined with a crown-rump length >10 mm with no evidence of heart pulsations on two separate occasions at least 7 days apart. Whenever there is uncertainty about the viability of a pregnancy, a repeat scan at an interval of 1 week is necessary.	10(90.9)
If on ultrasound the uterus is empty, there is adnexal mass, tubal ring, free fluid in the adnexa cul de sac area or complex fluid in the pelvis AND serum β-hCG value exceeds a discriminatory level of 1500–3000 mIU/mL, this is an indication for ectopic pregnancy. In addition, complete miscarriage should be considered if serum β-hCG is falling.	11(100)
Proper management and control of diabetes and hypothyroidism if identified during pregnancy, should be addressed in prone women with threatened miscarriage in the first trimester.	11(100)
It is recommended that patients start or continue to take prenatal vitamins with folic acid supplementation. A daily supplemental dose of 400 μg/day of folic acid is recommended.	11(100)
Alloimmunization prevention by the administration of Rh(D) immunoglobulin should be considered for patients who have vaginal bleeding in the setting of pregnancy in a patient who is Rh-. It has been suggested that a 50 mcg dose of immunoglobulin is effective at alloimmunization prevention up to and through the 12th week of gestation, however, it is considered acceptable to give the standard 300 mcg dose due to non-availability of smaller dose.	11(100)
Anti D prophylaxis should be offered to women with threatened miscarriage of less than 12 weeks gestation if the bleeding is recurrent, heavy and associated with abdominal pain.	11(100)
Bedrest and other activity restrictions have not been found to be efficacious in the prevention of a threatened miscarriage progressing to spontaneous abortion and have been shown to increase the risk of other complications including deep vein thrombosis and/or pulmonary embolism and therefore should not be recommended.	10(90.9)
Progesterone and human chorionic gonadotropin (hCG) are most commonly prescribed for women with threatened miscarriage.	10(90.9)
Progesterone is advised to females who have experienced one or more prior pregnancy losses and have bleeding early in the current pregnancy.	9(81.8)
The available evidence suggests that Folic acid supplements are essential.	11(100)
Apart from Folic acid, routine vitamin B supplementation in females for threatened miscarriage is not essential.	11(100)
Supplementation with low dose of vitamin D seems to be beneficial for threatened miscarriage, and a randomized double-blinded study showed that supplementation with vitamin D3 (400 IU/day) led to a decreased incidence of miscarriage.	11(100)
Apart from folic acid, taking vitamin supplements in early pregnancy does not prevent miscarriage.	11(100)

The expert panel focused on monitoring or expectant handling with no recommendation on medicinal or surgical procedures at this stage. Furthermore, they emphasized the importance of follow-up especially with excessive bleeding, pain, fever, or abdominal cramps. They also indicated the need for ultrasound follow-up and agreed that the timeline to be between seven and 10 days in clinically stable or asymptomatic patients until viable pregnancy or inevitable abortion is confirmed.

However, the panel agreed that to help ease the pain of cramping, analgesia can be given but non-steroidal anti-inflammatory drugs (NSAIDs) should be avoided. The panel also agreed that 50mcg of immunoglobulin is effective in alloimmunization prevention up to and through the 12th week of gestation. Besides, they agreed to give progesterone and human chorionic gonadotropin (hCG) as they are frequently administered to pregnant women who are at risk of threatened miscarriage. The statements reported and agreed upon reached consensus after much discussion indicating the need for further studies in this field.

Education and awareness

Statements related to the value of education and awareness are outlined in Table 7. Experts (100%) agreed that women should be educated about the adverse effects of smoking, drug, and alcohol use on pregnancy outcomes. Moreover, being aware of these factors, screening for them, and educating women about these factors were deemed necessary by all the experts. They summarized these points into four statements which were unanimously agreed on.

**Table 7:** Level of agreement on education and awareness statements

Statement	Level of agreement (%)
Women should be educated about reducing the risk of traumatic events and should be screened for risk for intimate partner violence.	11(100)
Women should be educated about the adverse effects of smoking, drug, and alcohol use on pregnancy outcomes.	11(100)
It is preferred to advise patients to avoid strenuous activities and sexual activity to maintain pelvic rest at least until the cessation of vaginal bleeding.	11(100)
It is reasonable to recommend preconception counseling to identify and modify risk factors.	11(100)

## Discussion

Risk assessment

Women presenting with pregnancy can be assessed for threatened miscarriage for risk factors. Some risk factors are unmodifiable or uncontrollable while others are modifiable and can be prevented or treated [12,15]. General risk factors: One of the most recognized risk factors for threatened miscarriage is maternal age. The association between age and threatened miscarriage risk has a J-shaped curve pattern. Women younger than 20 years had an odds ratio of 1.7 and those over 40 years had an odds ratio of 1.4 [12].

Similarly, miscarriage risk and age also form a J-shaped curve pattern. The age group with the lowest risk for miscarriage is between 25 and 29 (9.8%) while the highest is in women 45 years of age or older (53.6%) [16]. Iqbal et al. studied retrospectively 517 Pakistani pregnant women in Karachi for risk factors of spontaneous abortion. This study showed that women over 40 years of age were 10 times more likely to have a miscarriage as compared to those younger than 30 years of age (OR=10.28, 95% CI:3.83-27.61, p=0.001) while the risk increased for those between 30 and 35 years of age but was not statistically significant [17]. Another study indicated that the age group of 30-34 years had an odds ratio of 2.22 while the age group of 35 and older had an odds ratio of 4.59 of having a miscarriage [18]. Moreover, being older than 34 years of age is a risk factor for threatened miscarriage specifically [19,20]. Advanced maternal age is associated with chromosomal abnormalities which might by itself be a reason for threatened miscarriage [20,21]. Hereby, the expert panel has acknowledged age as a risk factor for threatened miscarriage and proposed a change of age limit to 35 and older for ease of abiding by the widely used advanced maternal age limit.

Additionally, previous history of threatened miscarriage can also be considered as a risk factor. The risk of threatened miscarriage increases by around 2.2 times in those who had a previous miscarriage. Iqbal et al.'s study on Pakistani women also showed that pregnant women with a history of miscarriage were around three times more likely to have a miscarriage than those with no history [17].

Other studies from different populations showed an incremental effect of previous miscarriage numbers on the recent pregnancy outcome [15,16,22]. While the history of one previous miscarriage increased the risk of miscarriage in the current pregnancy by 54%-84%, in the case of two previous miscarriages this risk was increased [16,23]. Thus, all panelists agreed to consider the history of two or more miscarriages as a risk factor in accordance with published studies [19,20]. 

In regard to trauma, direct or strong trauma during pregnancy might have a devastating effect in late pregnancy but is usually uncommon in early pregnancy. Moreover, nine out of 10 injuries during pregnancy are minor [23]. A study in Kuwait showed that most of the minor traumas were experienced by women in the first trimester, who were all wearing seat belts [24]. Besides motor vehicle accidents, the leading causes of obstetric trauma include falls, assaults domestic violence, burns, poisoning, and penetrating trauma [25]. Minor trauma is responsible for 60%-70% of fetal loss. The most common cause of fetal death following trauma is placental abruption. Placental abruption usually occurs during the second and third trimesters. The thick-walled uterus is still protected by the pelvis during the first trimester and the chances of injury are little [24]. Furthermore, the experts disagreed initially about trauma as a risk of threatened miscarriage during the first trimester. However, following discussing statements and evidence, they agreed unanimously that direct and severe trauma to the lower abdomen might have detrimental effects.

Health-related risk factors

The statements related to health risk factors are outlined in Table 1. Vaginitis, cervicitis, or a cervical polyp can cause vaginal bleeding or threatened miscarriage [26]. Immunological dysfunction and infection which can be bacterial, viral, parasitic, or fungal [20]. Infection with genital herpes simplex, human immunodeficiency virus (HIV)-1 and 2, and group B streptococci increased the likelihood of threatened miscarriage [19]. Around 7.84% of pregnant women who had urinary tract infections caused by Escherichia coli experienced threatened miscarriage [27]. A study of Pakistani pregnant women reported a risk of threatened miscarriage in malaria-infected women despite being a low percentage [28]. However, experts considered the increased likelihood because of malaria being common in Pakistan. Similarly, pregnant women infected with dengue fever had an increased risk of threatened miscarriage and miscarriage [29]. Recently, a cohort study on Japanese pregnant women compared the before, during, and after COVID-19 epidemic periods and found no statistical difference in risk for threatened miscarriage between them [30]. Literature is scarce with regard to COVID-19-positive pregnant women who are in the first trimester. A few studies reported negative consequences of COVID-19 on maternal and fetal outcomes [31-33]. Considering the coagulation disorder associated with COVID-19, a cautious approach was advised by the panelist [34].

Furthermore, women who have a pre-existing medical condition are more likely to have threatened miscarriage than those who have no medical condition [12,35]. Of these medical conditions, endocrinological conditions and thrombophilia are acknowledged as risk factors. Several endocrinological factors have been associated with early miscarriage including poorly controlled diabetes, polycystic ovary syndrome, and thyroid disease [19,20,36]. Zhang et al. indicated that women with untreated subclinical hyperthyroidism had a higher prevalence of early miscarriage as compared with euthyroid pregnant women [37]. Moreover, women with pregestational diabetes had a higher frequency of spontaneous miscarriage and congenital defects. Pregnancies with poor glycemic control showed a five-fold increase in the total pregnancy loss rate as compared to controlled diabetes cases [38]. All experts agreed on the importance of including these endocrinological disorders as risk factors for threatened miscarriage.

Thrombophilia can be a hereditary or acquired condition that increases the risk of thrombosis. Pregnancy itself increases the risk of thrombosis. Only specific individualized testing is recommended in pregnant women. Thrombophilia can cause recurrent pregnancy loss, but absolute risk remains low. No data were reported concerning vaginal bleeding or threatened miscarriage [39-42]. With a lack of extensive literature and specific recommendations concerning threatened miscarriage, the expert panel opted for considering acquired thrombophilia to be a risk factor while the association with inheritable thrombophilia effect was deemed unclear.

Lifestyle-related risk factors

Statements related to lifestyle risk factors are outlined in Table 2. Several lifestyle factors might influence the risk of threatened miscarriage and patients can be advised to modify it [12]. Weight and obesity are well-known risk factors for threatened miscarriage and miscarriage [43,44]. Pakistani pregnant women showed an increased risk of miscarriage if underweight or overweight/obese as compared to normal-weight women [17].

Other well-known lifestyle risk factors include smoking and the use of illicit drugs [20]. Not only smoking but also second-hand smoking increases the risk of threatened miscarriage. Second-hand smoking increased the threatened miscarriage risk with OR=2.93 (95% CI 1.32-6.48) [45].

Also, excessive caffeine and alcohol intake increases the risk of threatened miscarriage [20]. The consumption of caffeine will increase the risk of threatened miscarriage by around two-fold [12,44]. Alcohol consumption of 2-3.5 drinks per day results in an increased risk of miscarriage in the first trimester [15]. The experts agreed to consider excess weight, underweight, smoking, caffeine, alcohol, and illicit drug use as risks for threatened miscarriage with dose-related factors for smoking, caffeine, and alcohol consumption.

Diagnosis of threatened miscarriage

The diagnosis of threatened miscarriage is made by physical examination, imaging approaches, and laboratory tests [46].

Physical examination

Statements related to physical examination to diagnose threatened miscarriage are outlined in Table 3. Physical examination aims to identify the various elements noted in the previous sentence in order to diagnose or rule out threatened miscarriage. The characteristic symptom is vaginal bleeding with or without abdominal pain. Physical examination is important to identify the source of bleeding and potential etiology. Alternatively, it also can help identify any extravaginal cause of bleeding or abdominal pain [47].

The physical examination usually includes three main components: an abdominal exam, pelvic exam, and internal vaginal exam along with a speculum exam. The Dutch guideline on threatened miscarriage for general practitioners recommended a set of exams during the first and follow-up visits with a recommendation to follow up after 10 days. The exams recommended for the first visit included abdominal, vaginal, and speculum examinations. The exams of the follow-up visit included vaginal and speculum examination. In case of continued blood loss, ultrasound is recommended [13].

Abdominal examination in pregnancy is important to identify the cause of acute abdominal pain. Acute abdominal pain can be related to pregnancy or can be of non-gynecologic etiology. The pregnancy-related etiologies might include threatened miscarriage, EP, molar pregnancy, and degenerative changes in a fibroid or complication in an ovarian cyst (torsion, infection, or rupture). The non-gynecologic etiologies commonly include any of the gastrointestinal causes. The different quadrants should be palpated starting with the one with the least pain because each quadrant might be indicative of a certain etiology [48].

The second recommended physical examination is a pelvic exam. Pelvic examination was not very beneficial in case of ultrasound availability to assess women presenting to the emergency department with potential miscarriages. However, the pelvic exam should be considered for acute cases to provide diagnostic information when ultrasound is not available [49]. Experts also agreed to include pelvic examination as a tool for differentiating between threatened and inevitable miscarriage and gestational age identification, but they paraphrased the consensus statement to have a conditional phrase of “if facility ultrasound is not available.” 

In case of threatened miscarriage, the internal vaginal examination along with the use of a speculum can be beneficial. The vaginal examination can show if the cervical os is closed and no cervical motion tenderness is found. Although the presence of closed os can indicate threatened miscarriage it remains not a confirming diagnosis [50]. Clinical judgment was not a valid replacement for ultrasound assessment in diagnosing threatened miscarriage; however, the multi-logistic analysis showed that the presence of blood at speculum examination and not passing blood clots are negatively associated with a viable pregnancy [51]. The experts agreed on several statements concerning the vaginal and speculum examination all unanimously.

In addition to vaginal and speculum exams, sometimes it would be beneficial to ensure that infection is not the reason for the threatened miscarriage. Infection is considered one of the risk factors as indicated above. The diagnosis of infection can be confirmed by a vaginal swab or pap smear [52]. However, the experts wanted to ensure the use of this tool for assessing vaginal infection only in those with the suspected cases and agreed that a “high vaginal swab for culture sensitivity is recommended in the presence or suspicion of vaginal infection.”

The ultrasound visualization of open internal os indicates inevitable miscarriage, as does the sac being low within the uterus. Also, if cervical dilatation is noted during physical or ultrasound examination, a threatened miscarriage proceeding eventually to complete miscarriage becomes unavoidable [52].

Imaging approaches

Statements related to imaging approaches to diagnose threatened miscarriage are outlined in Table 4. The gold standard for assessing bleeding in early pregnancy is TVS for both diagnosis and follow-up monitoring [53,54]. The ubiquitous availability, reasonably low cost, and capacity to capture high-resolution images in real-time and at the same time being noninvasive, safe, and without hazards of radiation are all benefits of ultrasound imaging [55]. It is especially helpful in individuals who are bleeding and have a positive pregnancy test, but their intrauterine pregnancy has not yet been verified by imaging.

The experts agreed that TVS is recommended in checking for structural abnormalities of the uterus, evidence of viability and intrauterine location, the possibility of extrauterine pregnancy or heterotopic pregnancy, and loss of one of the multiple gestations. One of the experts emphasized the importance of the determination of chronicity in the case of multiple gestations [56].

Concerning viability, the fetal heart activity and the discrepancy between gestation age and crown-to-rump length were emphasized. Ultrasound has improved diagnosis by rapid confirmation of viability [19]. Various studies have suggested that a viable baby can be seen at 3.0 to 5.3 mm Crown-rump length (CRL) [57]. A CRL of 6 mm or greater has been proven to have a 100% success rate in identifying a viable pregnancy [56]. When a heartbeat is absent with a CRL of less than 7.0 mm or a mean gestational sac diameter of less than 25.0 mm determined by a single ultrasound, a repeat scan is advised within seven days if using transvaginal ultrasound or 14 days if using transabdominal ultrasound [56,58]. The risk of miscarriage is significantly increased by the interplay of various factors. For instance, logistic regression analysis revealed that the likelihood of miscarriage in women who were bleeding between weeks 5 and 12 of pregnancy was 84% in cases of fetal bradycardia when coincides with discrepancies between the CRL and the diameter of the gestational sac, as well as menstrual and sonographic age; this risk was reduced to 6% if none of these factors were evident [59,60].

When a woman is experiencing a threatened miscarriage, ultrasound is crucial in identifying prognostic indicators for a poor outcome, such as a short CRL and an empty gestational sac [61]. Anembryonic pregnancy or early embryonic death can be diagnosed when ultrasound imaging shows an intrauterine gestational sac, embryonic crown-rump length ≥7 mm without cardiac activity or the diameter of the gestational sac is ≥25 mm without a yolk sac or embryo [53,62]. A statement was added concerning anembryonic pregnancy. Anembryonic pregnancy was confirmed by gestational sac ≥25 mm without a yolk sac or embryo on TVS.

To ascertain if the pregnancy in these patients is intrauterine or extrauterine EP and, if intrauterine, whether it is viable or not, an ultrasound examination is carried out [52,53,63,64]. It is important to note that the diagnosis of EP should not be based on an inability to visualize an intrauterine pregnancy, but rather on the positive visualization of an adnexal mass separately from the ovaries on TVS images [42]. This was another point emphasized in the form of a statement by the expert panel.

Pedersen et al. demonstrated in their study that subchorionic hematomas are common with a prevalence of 18% in women who present with a threatened miscarriage [65]. These hematomas are insignificant sonographic findings in patients with vaginal bleeding in weeks 9-20 of pregnancy, as they are not associated with adverse pregnancy outcomes [66].

The use of ultrasound in clinically diagnosed threatened miscarriages may help doctors make a firm diagnosis so that the patients can receive the appropriate care they need [67]. The panel agreed that Sonography/ultrasound examination is the cornerstone to check heartbeats and if there is fetal bradycardia then they should be referred to a specialist. Since the prognostic value of a subchorionic hematoma in ultrasound has been disputed, there was a unanimous agreement that it has no association with threatened miscarriage. All other statements were undertaken during the discussion and agreed upon with the majority being approved unanimously.

Laboratory tests

Statements related to the value of laboratory tests are outlined in Table 5. The most important laboratory test is serum beta hCG along with complete blood count, blood group Rh factor, and clotting profile if indicated.

hCG testing is recommended for pregnant patients who are experiencing pain and/or bleeding rather than urine testing. Because there is a large range of normal values at each week of pregnancy, it is impossible to determine if a pregnancy is normal based on a single hCG level [68]. When determining whether an early pregnancy is normal or abnormal, trends in β-hCG levels are helpful. The level of β-hCG above which a transvaginal ultrasound should reveal an intrauterine pregnancy is known as the discriminatory level (1,500 to 3,000 mIU/mL). When an intrauterine pregnancy cannot be detected and hCG levels are over the discriminatory level, early pregnancy loss or an EP would be suspected [69-71]. The experts all agreed that it is not useful to check hCG concentration once the presence of an intrauterine pregnancy has been established sonographically. However, they recommended serial measurements of hCG during the first six weeks of pregnancy if ultrasonography is not conclusive of diagnosis.

Puget et al. found out that for 41% of women with pregnancies of uncertain viability, serial hCG offers a quick, reproducible way to determine if a pregnancy is viable. Results can be obtained in just two days as opposed to the usual 7-14 [72]. Serial hCG concentration measurements are recommended to distinguish between normal and abnormal pregnancies [73-76].

For women experiencing first-trimester bleeding, pain, or both, serial hCG measurements are frequently used. However, they are unable to determine the location of the gestational sac. The concentration of β-hCG in the first trimester of a healthy pregnancy rises quickly, doubling every two days on average. Viability has been defined as an increase of at least 66% over the preceding 48 hours [77-80]. Thus, the statement was agreed upon level of 1,500 and 2,000 IU/mL considering an acceptable range when considered alongside ultrasound to diagnose EP or failure of intrauterine gestational sac.

Pregnancy-associated protein-A (PAPP-A) and other hormone assays, such as progesterone, estrogen, and inhibin A, were found to be less helpful [81]. The issue of biochemistry evaluations for β-hCG, free β-hCG, Bioactive/immunoreactive ratio hCG, progesterone, Inhibin-A, and CA125 took a lot of debate and they all agreed that in a country like Pakistan where such tests are either inaccessible or expensive and when the healthcare system in Pakistan does not financially support, such tests should not be routinely offered as patients cannot afford them. Thus, the panel agreed to keep only the serum β-hCG as a standard investigation.

It is important to note that all hemodynamically unstable women are recommended to do coagulation studies and hemoglobin. Women with heavy vaginal bleeding are advised to have a baseline hemoglobin measurement in hemodynamically stable patients, especially if the bleeding is persistent [82]. In order to assess the level of blood loss and status of anemia a widely available complete blood count test was recommended by the expert panel, which is usually routinely done for blood loss and anemia. In addition, pregnant females should perform Rh factor testing if their Rh status is not known at the time of presentation [68,83]. The panel stated that in Pakistan there is overuse of blood thinners so only women with recurrent miscarriage can be offered thrombophilic blood screening and be referred to a specialist. The tests are expensive, but the overuse of blood thinners will cost more and will be a waste.

Management of threatened miscarriage in the first trimester

Statement related to the management of threatened miscarriage are outlined in Table 6. The management of threatened miscarriage at the initial stages is based on follow-up. As per the Dutch guideline, it was recommended pregnant women with bleeding to follow up after 10 days and even earlier, if they noted changes in bleeding or low abdominal pain or fever [13,84]. A similar statement was adopted by the expert panel focused on monitoring or expectant handling with no recommendation on the medicinal or surgical procedures at this stage. Furthermore, they emphasized the importance of follow-up especially with excessive bleeding, pain, fever, or abdominal cramps. They also indicated the need for ultrasound follow-up and agreed that the timeline to be between seven and 10 days in clinically stable or asymptomatic patients until viable pregnancy or inevitable miscarriage is confirmed.

NSAIDs use around conception was associated with an increased risk of miscarriage with a dose-response relationship [85]. Thus, the panel agreed that to help ease the pain of cramping, analgesia can be given but NSAIDs should be avoided in line with research findings [86].

Certain ultrasonography findings, such as bradycardia at more than seven weeks of gestation, a small sac in comparison to the embryo's size enlarged with a subchorionic hematoma, and an irregularly shaped (crenelated) yolk sac are linked to early pregnancy loss [58,87-93]. On ultrasonography, an EP is suspected if the uterus is empty, there is an adnexal mass, a tubal ring, free fluid in the adnexa cul de sac area, or complex fluid in the pelvis, and the serum hCG level is higher than the discriminating range of 1,500-3,000 mIU/mL [94,95].

A missed miscarriage was defined as an embryo with a crown-rump length > 10 mm and no signs of a heartbeat on two different occasions that were at least seven days apart. Before a conclusive diagnosis can be made, a scan should be repeated at an interval of one week to confirm the viability of a pregnancy [96,97]. If serum hCG levels are dropping, a complete miscarriage should be taken into consideration [98].

Rh-ve patients who experience vaginal bleeding during pregnancy should be given Rh(D) immunoglobulin as alloimmunization preventive therapy [83]. The panel agreed that 50mcg of immunoglobulin is effective in alloimmunization prevention up to and through the 12th week of gestation, however, it is considered acceptable to give the standard 300 mcg dose due to non-availability of a smaller dose in accordance with published research [99].

The National Institute for Health and Care Excellence (NICE) has advised that progesterone be given to women who suffer bleeding in the first trimester of pregnancy and have previously experienced a miscarriage [100]. In accordance with published data, the panel agreed to give progesterone and hCG as they are frequently administered to pregnant women who are at risk of threatened miscarriage.

The continuing pregnancy success rate was greater in women who took dydrogesterone treatment compared to women who got conservative treatment, with a significant difference (p=0.037). The experts agreed on 40 mg of dydrogesterone as a starting dose, then 10 mg twice daily for a week in the first trimester was given when having vaginal bleeding, in accordance with published studies [101,102].

Bedrest and other activity restrictions are not effective in preventing a threatened miscarriage from developing into a spontaneous miscarriage. Thus, the expert agreed that bed rest and restrictions have been shown to increase the risk of other complications such as deep vein thrombosis and/or pulmonary embolism, and should not be advised in line with published studies [103].

In pregnant women who are at risk of threatened miscarriage in the first trimester, proper management and control of diabetes and hypothyroidism should be addressed [104]. It is advised that patients begin or continue taking prenatal vitamins with folic acid supplementation at a daily dose of 400 mcg to prevent neural tube defects [105-107]. The information that is now available indicates that Iron supplements are required for pregnant women, however, excessive dosage should be avoided [108-110]. Apart from folic acid, taking vitamin supplements in early pregnancy does not prevent miscarriage [111]. It is not advised to take zinc and/or copper supplements when pregnant as cases of threatened and spontaneous miscarriage had greater Copper levels in their serum [112]. Early-stage zinc deficiency during pregnancy has been linked to congenital abnormalities and threatened miscarriage [113]. The advantages and disadvantages of routine vitamin-B supplementation in women with threatened miscarriage are not sufficiently supported by research [20]. For women who are at risk of threatened miscarriage, vitamin D supplementation appears to be beneficial [114]. A randomized double-blinded trial found that women who took vitamin D3 (400 IU/day) had a decreased rate of miscarriage [115]. The statements reported and agreed upon reached consensus after much discussion indicating the need for further studies in this field (Table 6).

Education and awareness

Statements related to the value of education and awareness are outlined in Table 7. Studies have confirmed that drinking alcohol during pregnancy increases the risk of miscarriage. In comparison to women who abstained, several studies discovered that women who drank four or more drinks per week had a twofold higher chance of miscarriage [116,117]. In Ness et al. study population, cocaine, and tobacco use were widespread and significantly increased the incidence of spontaneous miscarriage. Smoking (16%) and cocaine (8%) combined would be responsible for 24% of the spontaneous miscarriages among women and adolescents [118]. Thus, the panel agreed that women should be educated about the adverse effects of smoking, drug, and alcohol use on pregnancy outcomes.

Patients should be encouraged to refrain from physically demanding activities and maintain pelvic rest at least until vaginal bleeding stops [119,120]. In addition, intimate partner violence risk assessment and education are important to lower the risk of traumatic incidences in women [19,121-123]. Being aware of these factors, screening for them, and educating women about these factors were deemed necessary by the expert committee (Table 7). They summarized these points into four statements which were unanimously agreed on.

Strengths and limitations

This study is the first of its kind in the Pakistani population dealing with threatened miscarriage; however, this study was not without certain limitations and also some strengths. The panel consisted of 11 experts which was less than the recommended “12 experts” [124]. On the other hand, the response rate was very high and complete during each step of the Delphi process.

The meeting was conducted in person and virtually which allowed for more feedback and higher participation. This was an inherent limitation that allowed us to conduct the meeting during the COVID-19 restrictions. On the other hand, virtual meetings might have resulted in the loss of anonymity and might have led to a higher “dominant individuals” effect [125]. Additionally, the purposive sampling technique was used in our study which ensured adequate group dynamics during the meeting, but it might introduce bias, as it does not guarantee a representative sample of all relevant experts [126].

## Conclusions

Only a few studies assessed the risk factors, diagnosis, and management of threatened miscarriage in Pakistani women. Thus, this Delphi-based study helps combine the available evidence worldwide and the clinical experience of local experts to devise a way to optimize the assessment, diagnosis, and management of threatened miscarriage in Pakistan. This consensus is comprehensive because it includes all aspects from risk factor assessment, diagnosis, and evaluation as well as management of threatened miscarriage. Further studies can validate the statements agreed on by the expert panel. Also, this consensus can be a means for involving other key players such as general practitioners, health communicators, and pregnant women. These key players might add to the statements, assessing the feasibility and benefits of this consensus or help in giving different perspectives to the application of the statements. 
